# Evaluation of Alere i RSV for Rapid Detection of Respiratory Syncytial Virus in Children Hospitalized with Acute Respiratory Tract Infection

**DOI:** 10.1128/JCM.02433-16

**Published:** 2017-03-24

**Authors:** Rebecca Marie Peters, Sarah Valerie Schnee, Julia Tabatabai, Paul Schnitzler, Johannes Pfeil

**Affiliations:** a Center for Childhood and Adolescent Medicine (General Pediatrics), University Hospital Heidelberg, Heidelberg, Germany; b German Center for Infectious Diseases (DZIF), Heidelberg partner site, Heidelberg, Germany; c Center for Infectious Diseases, Virology, University Hospital Heidelberg, Heidelberg, Germany; Boston Children's Hospital

**Keywords:** Alere i RSV, pediatric infectious disease, point-of-care, rapid tests, respiratory syncytial virus, respiratory tract infection

## Abstract

Alere i RSV is a novel rapid test which applies a nicking enzyme amplification reaction to detect respiratory syncytial virus in point-of-care settings. In this study, we evaluated the Alere i RSV assay by using frozen nasopharyngeal swab samples that were collected in viral transport medium from children hospitalized with acute respiratory tract infection during the 2015-2016 winter season. Alere i RSV assay results were compared to those for Altona RealStar RSV real-time reverse transcription-PCR (RT-PCR). We found that the overall sensitivity and specificity of the Alere i RSV test was 100% (95% confidence intervals [CI], 93% to 100%) and 97% (95% CI, 89% to 100%), respectively. Positive samples were identified within 5 to 7 min from sample collection. Overall, the Alere i RSV test performed well compared to the RT-PCR assay and has the potential to facilitate the detection of RSV in point-of-care settings.

## INTRODUCTION

Respiratory syncytial virus (RSV) is the most important cause of acute respiratory tract infection (aRTI) in neonates and young children (1). RSV infection leading to bronchiolitis or pneumonia can be fatal, especially in children and older adults at risk due to conditions like premature birth, chronic lung disease, congenital heart disease, or immune deficiency (2).

Pediatricians who admit a child with aRTI to inpatient care are usually unaware of the underlying respiratory pathogen (3). Rapid point-of-care diagnostic tools allow timely identification of viral pathogens. The availability of sensitive rapid RSV tests is critical to optimize care management, minimize unnecessary antibiotic use, and provide targeted infection control for children hospitalized with RSV infection.

A major limitation of point-of-care RSV testing is the low sensitivity of currently available rapid antigen detection tests (RADT). In general, RADT sensitivity is strongly dependent on high viral load of respiratory specimens and therefore performs best in young infants with classical symptoms of RSV bronchiolitis. A pooled RADT sensitivity of 80% (95% confidence interval [CI], 76% to 83%) was reported for pediatric patients (4). In our screening population of children <18 years of age who were hospitalized with any symptom of respiratory tract infection, we observed RADT sensitivity of 63% (95% CI, 55% to 72%) compared to reverse transcription-PCR (RT-PCR) (5), while in adults, sensitivities of different RADT were <25% (6). As an alternative to RADT, rapid nucleic acid amplification assays have been developed to facilitate early recognition of RSV infection. These assays provide high sensitivities but require a testing time of at least 1 h (7, 8).

The Alere i system is a rapid, automated *in vitro* diagnostic test which utilizes a nicking enzyme amplification reaction. The platform technology was previously developed for the detection of influenza virus (Alere i influenza) and provides test results in less than 15 min. The test is optimized for point-of-care settings and does not require specific training or experience in laboratory techniques. A number of studies have evaluated this rapid influenza test in comparison to both viral culture and RT-PCR, reporting sensitivities between 45 and 100% (9–15). Recently, the platform has been extended with test cartridges for the detection of streptococcus A (Alere i Strep A) and RSV (Alere i RSV). Both assays are being introduced into routine clinical care. No data have been published regarding the sensitivity and specificity of the novel Alere i RSV test. The objective of our study was to evaluate the performance of the Alere i RSV test for rapid detection of RSV in children hospitalized with acute respiratory tract infection.

## RESULTS

A total of 117 nasopharyngeal swab (NPS) samples were included in our study and tested by Alere i RSV and Altona RealStar RSV RT-PCR. In three samples, the Alere i RSV assay was invalid, as the lyophilized test reagents were not properly dispensed due to a handling failure. These samples were excluded from the analysis.

The 114 remaining NPS were collected from patients hospitalized due to lower respiratory tract infection (*n* = 79), upper respiratory tract infection (*n* = 12), or nonrespiratory diagnoses, including, e.g., febrile convulsions or gastroenteritis with concomitant respiratory tract infection (*n* = 14). In 9 patients, the admission diagnosis was not reported. The median age of the patients included in our analysis was 12 months (range, 2 weeks to 17.7 years).

Altona RealStar RSV RT-PCR was RSV positive in 43% (49/114) and RSV negative in 57% (65/114) of cases. RSV A and B was detected in 20 (41%) and 29 (59%) of RSV-positive samples, respectively. The mean threshold cycle (*C_T_*) value of RSV-positive samples was 22.7 (95% CI, 21.4 to 24.0), and the median *C_T_* value was 22.3 (range, 15.0 to 33.8). Mean *C_T_* values did not differ between RSV A- and RSV B-positive samples (RSV A, 23.2; RSV B, 22.5; *P* = 0.6).

**FIG 1:**
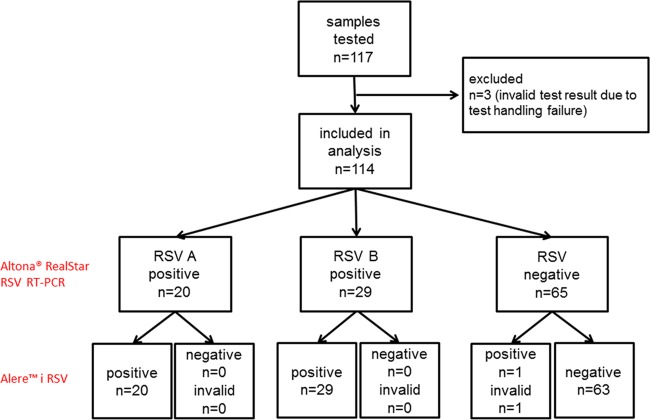
Study flow chart.

The Alere i RSV test result was true positive in 49 out of 49 samples and true negative in 63 out of 65 samples. We did not undertake comprehensive analytical specificity testing but note that the 65 samples with a negative Altona RealStar RSV RT-PCR result included samples which had previously tested positive for influenza A (*n* = 10), influenza B (*n* = 6), parainfluenza (*n* = 3), human metapneumovirus (*n* = 9), coronavirus (*n* = 7), rhinovirus (*n* = 17), parechovirus (*n* = 1), and adenovirus (*n* = 1). Of the 2 samples with diverging test results, 1 test result was interpreted as false positive, whereas the other test result was invalid due to sample interference (Fig. 1). In both cases, we could not conduct confirmation testing due to limited sample volume. For the calculation of the Alere i RSV specificity, both samples with diverging test results were considered false positive. We thus report a conservative estimate of the Alere i RSV performance.

The combined RSV A and B test sensitivity for the Alere i RSV was 100% (95% CI, 89 to 100%). The specificity of the Alere i RSV test was 97% (95% CI, 89% to 100%) (Table 1).

**TABLE 1:** Comparison of the Alere i RSV assay to the Altona RealStar RSV RT-PCR

Alere i RSV^*a*^ result	Altona RealStar RSV RT-PCR result	
	Positive	Negative
Positive (*n*)	49	2^*b*^
Negative (*n*)	0	63
Alere i RSV sensitivity [% (95% CI)]	100 (93–100)	
Alere i RSV specificity [% (95% CI)]	97 (89–100)	

a The Alere i RSV assay displays a combined result without providing further information on RSV subtype.

b Including one sample with invalid Alere i RSV test result.

The Alere i RSV test includes a 3-min step of preheating the lysis buffer, followed by the nicking enzyme amplification reaction with a maximum duration of 10 min. The assay identifies positive test results as soon as an amplification threshold is reached. In this study, the median duration of the nicking enzyme amplification reaction in RSV-positive samples was 1.8 min (range, 1.7 min to 3.9 min). Hence, in RSV-negative samples, the total duration of the test assay was 13 min (3 min of preheating plus 10 min of amplification), whereas it was only approximately 5 to 7 min in RSV-positive samples.

**FIG 2:**
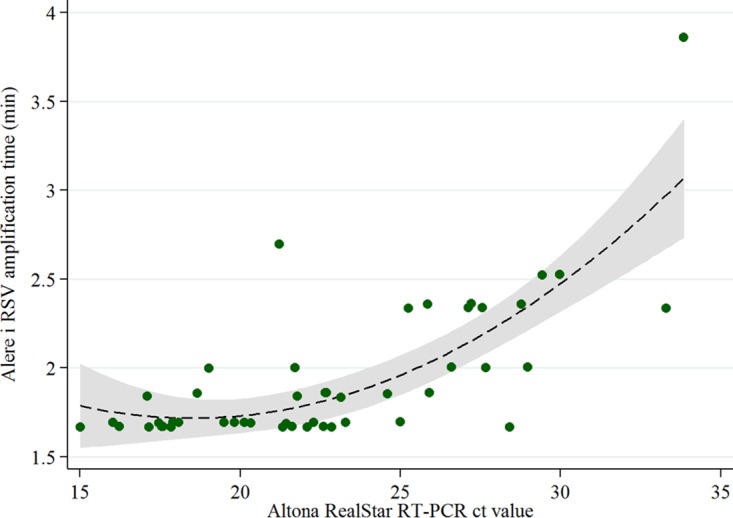
Alere i RSV amplification time versus Altona RealStar RSV RT-PCR *C_T_* value. Green dots represent individual samples. The dashed line depicts the predicted duration of amplification time with 95% CI (gray color).

Positive test results were identified quicker in samples with high viral load (*C_T_* value of <25), although the absolute differences are small and probably of minor importance in routine care (Fig. 2).

## DISCUSSION

In this study, we found that the novel Alere i RSV assay detects RSV infection with high sensitivity and specificity in NPS samples obtained from children hospitalized with acute respiratory tract infection. Results are delivered within 13 min, and positive samples usually are identified within 7 min. The assay displays a combined positive or negative test result for both RSV A and B without providing further information on RSV subtype. Both RSV A and B strains were reliably detected by the test assay. This provides clinicians with the confidence to act appropriately when a negative Alere i RSV test result is obtained. The assay requires 3 to 4 min of hands-on time. In our experience, it was user friendly and efficient, although some initial training is still required. We obtained 3 invalid test results due to a handling failure in the transfer of the sample eluate into the lyophilized test base.

Our study has two limitations. First, our analysis was limited in terms of sample size and was conducted on NPS collected at one pediatric hospital during a single winter season. The Alere i RSV assay performance might differ in other settings, particularly when samples with lower viral load are considered. Our study results therefore are not necessarily applicable to adult testing. Second, this study used frozen samples in a laboratory setting. As the Alere i RSV test is intended for point-of-care analysis, it will usually be conducted in a clinical rather than a laboratory setting. To corroborate our findings, future studies should be conducted with larger patient cohorts in the point-of-care setting.

We found the assay easy to use, and the operator is guided through the testing with immediate feedback in the case of incorrect handling. Furthermore, retesting of invalid samples, which was not feasible in our analysis, will usually be possible in point-of-care settings. We are therefore confident that the results reported in our analysis represent a realistic estimate of the Alere i RSV test performance for pediatric patients in point-of-care settings.

In summary, the novel Alere i RSV assay provides highly sensitive and specific test results in less than 13 min. It has the advantage of a considerably shorter test time than other currently available molecular test assays (7, 8) and a considerably higher sensitivity than any other currently available RADT assay (4). The Alere i RSV assay could become a valuable tool for rapid diagnosis of RSV infection in point-of-care settings.

## MATERIALS AND METHODS

### Patients and respiratory specimens.

During the 2015-2016 winter season (1 November 2015 to 1 April 2016), a nasopharyngeal swab (NPS) was obtained from children hospitalized with symptoms of acute respiratory tract infection at the Center for Childhood and Adolescent Medicine in Heidelberg, Germany.

NPS were collected in 1 ml universal transport medium (article number 806E013N; MSwab; Copan, Brescia, Italy) at the time of admission to inpatient care. After collection, all NPS specimens were refrigerated at 4°C. Within 24 h, aliquots were prepared and stored at −80°C for further analyses.

Demographic and clinical information were collected prospectively. Physicians were asked to specify a diagnosis on admission (i.e., reason for hospitalization) based on their clinical judgment. Diagnoses were classified to upper respiratory tract infection (URTI; including cases of rhinitis, pharyngitis, otitis media, and croup), lower respiratory tract infection (LRTI; including cases of bronchitis, bronchiolitis, and pneumonia), or other admission diagnoses (including, among others, cases of febrile convulsions, fever of unknown origin, and gastroenteritis).

### Alere i RSV and RT-PCR testing.

Frozen NPS samples were tested in parallel by Alere i RSV versus a CE-certified RT-PCR (Altona RealStar RSV PCR kit) as a gold standard. All NPS samples used in this study were thawed only once. For Alere i RSV testing, 200 μl of universal transport medium containing the NPS sample was added to the sample receiver containing 2.5 ml elution buffer. Two 100-μl volumes were transferred to the test base for isothermal amplification. For the Altona RealStar RSV PCR assay, viral RNA was extracted from 140 μl universal transport medium containing the NPS sample in 560 μl elution buffer using the QIAamp viral RNA minikit (Qiagen, Hilden, Germany). RNA was eluted in a total volume of 60 μl, of which 10 μl was transferred to the Altona RealStar RSV PCR master mix. RT-PCR results with a *C_T_* value of <35 were considered positive.

Alere i RSV log files were analyzed to report amplification time of all RSV-positive samples and to examine samples with invalid test results. Samples with invalid test results due to any kind of Alere i RSV test handling failure were excluded from the analysis. In contrast, samples with an invalid test result due to sample interference were considered false negative if the RT-PCR result was positive and as false positive if the RT-PCR reference was negative. Possible reasons for invalid test results include inhibition of the amplification reaction or delayed amplification in samples with low viral load. Notably, the manufacturer recommends retesting of samples with invalid Alere i RSV results. However, as the volume of the respiratory samples used in our study was limited to 400 μl, we were unable to follow this recommendation.

### Statistical analysis.

Statistical analyses were conducted using Stata/IC13.0 (StataCorp LP, College Station, TX, USA). Student's *t* test was used to compare mean *C_T_* values of RSV A-positive versus RSV B-positive samples. Quadratic prediction was applied to visualize the correlation between Alere i RSV amplification time and RT-PCR *C_T_* value.

### Ethical considerations.

This study was approved by the Ethical Research Board of the University Hospital Heidelberg, Germany (S-547/2015).
